# A PCR-based microwell-plate hybrid capture assay for high-risk human papillomavirus

**DOI:** 10.1007/s00705-014-2186-0

**Published:** 2014-08-05

**Authors:** Yumei Wang, Yan Liu, Yaping Ding, Nan Sun, Yafang Gong, Shangxian Gao

**Affiliations:** 1 Department of In Vitro Diagnostic Reagents, Key Laboratory of the Ministry of Health for Research on Quality and Standardization of Biotech Products, National Institutes for Food and Drug Control, No. 2 Tiantanxili, Beijing, 100050 China; 2 Shenzhen Kangmei Biotechnology Co., Ltd, Shenzhen, 518057 China; 3 Third Xiangya Hospital of Central South University, Changsha, 410013 China

## Abstract

Human papillomavirus (HPV) is associated with cervical cancer. In this study, we developed a high-throughput microwell-plate hybrid capture (MPHC) method for epidemiological studies of high-risk HPV (HRHPV). The results with 1238 cervical specimens from female outpatients showed a concordance rate of 94.3 % between the MPHC and Hybrid Capture II assay. The MPHC assay showed an average HRHPV rate of 29.3 % for high-risk populations in populous cities of China. The established MPHC assay could sensitively and specifically detect 13 types of HRHPV and is suitable for large-scale screening, especially in areas where real-time PCR or fluorescence equipment is unavailable.

**Electronic supplementary material:**

The online version of this article (doi:10.1007/s00705-014-2186-0) contains supplementary material, which is available to authorized users.

It is well established that persistent infection with high-risk human papillomaviruses (HPV) plays the leading etiological role in the development of cervical and anal cancer and their immediate precursors [1–3]. Based on sequence differences within their L1 gene, HPVs are divided into more than 100 different genotypes, in which approximately 40 types of HPV transmitted through the genital tract have been classified further as “low-risk” and “high-risk” based on their association with malignant lesions [4]. The worldwide prevalence of high-risk human papillomavirus (HRHPV) infection in women without cervical abnormalities ranges from 0 to 48.4 %, and in developing countries, such as the countries of Asia and sub-Saharan Africa, the prevalence is higher than that in developed countries [5–7]. China is currently estimated to have 130,000 new cervical cancer cases each year, accounting for 28 % of the world’s total [8]. Therefore, a high-throughput assay is urgently needed for assessing the level of the HRHPV epidemic in current populations for the diagnosis and treatment of cervical cancer [9–11].

Several HPV detection methods, including PCR-based methods and the Hybrid Capture II-based system, have been established to assess the presence of 13 high-risk HRHPVs [12–14]. In these methods, several consensus or degenerate primer pairs have been designed to amplify the relatively conserved region of the L1 gene in the HPV genome, including GP5/GP6 and PGMY09/PGMY11 [15, 16]. Although these consensus or degenerate primers could amplify a wide spectrum of HPV genotypes, the sensitivity and specificity of different primer sets to detect HPV DNA need to be evaluated in a clinical study, and the overall prevalence of HPV needs to be properly estimated by a variety of detection methods [16, 17]. Moreover, neither cytology-based screening nor molecular tests for HRHPV are widely available in developing countries, which lack the necessary human, financial, and material resources, including instruments such as real-time PCR equipment and fluorescence detectors. Therefore, it is essential to develop a low-cost, high-throughput screening method to test large numbers of patients in developing countries [18]. In this paper, we describe a sensitive, low-cost, high-throughput and easily operated microwell-plate hybrid capture (MPHC) assay that detects 13 HRHPV types using a novel set of primers that specifically bind to the L1 region.

For the MPHC system, the YR8/YR10 primer set was used, which contains eight forward primers and nine reverse primers. Both primer pools were conjugated with biotin at their 5′ ends. Two segments of the amplified sequence, one each from the sense and antisense strands, were selected as the capture probes. Each probe had an oligo-T linker modified with an amino group at its 5′ end. The sequences of all the primers and the probes and their GenBank accession numbers are shown in Table 1. Twenty-six HPV probe mixtures were immobilized to activated microwells. Compared with the PGMY11/PGMY09 consensus primer set, which produces a product of 450 bp [16], the YR8/YR10 primer set produces a shorter product of 185 bp, which reduces the spatial obstacles to hybridization, thus improving the overall efficiency of hybridization.

**Table 1:** Primers and probes used for the MPHC assay of HRHPV

Primer/probe	Name	Sequence (5′–3′)	Accession number and nucleotide position
Forward primer	YR8-A	Bio-GCACAGGGTCATAATAATGGTATTTGTTGG	gb|FJ202006.1|: bp4-33
	YR8-B	Bio- GCACAGGGACATAATAATGGCATTTGCTGG	gb|U12488.1|: bp1-30
	YR8-C	Bio-GCACAGGGCCACAATAATGGTATTTGTTGG	dbj|AB889493.1|: bp6587-6616
	YR8-D	Bio-GCACAGGGTCATAACAATGGTATTTGCTGG	gb|KF225496.1|: bp149-178
	YR8-E	Bio-GCTCAGGGTTTAAACAATGGTATATGTTGG	gb|KC470266.1|: bp6551-6182
	YR8-F	Bio-GCCCAGGGCCACAACAATGGTATATGTTGG	gb|KC470239.1|: bp6612-6641
	YR8-G	Bio-GCCCAGGGACATAATAATGGCATTTGTT	emb|AJ620205.1|: bp6696-7 = 6723
	YR8-H	Bio- GCACAGGGTCATAACAATGGTATCTGCTGG	gb|KC470221.1|: bp6532-6561
Reverse primer	YR10-A	Bio-TGAAAAATAAACTGTAAATCATATTCCTC	dbj|AB889494.1|: bp6767-6739
	YR10-B	Bio-TGAAAAATAAACTGTAAATCATATTCTTC	gb|KC991279.1|: bp146-118
	YR10-C	Bio-TGAAAAATAAACTGTAAATCATACTCTTC	gb|EF202168.1|: bp6626-6598
	YR10-D	Bio-TGAAAAATAAACTGTAAATCAAACTCCTC	gb|KC815977.1|: bp182-154
	YR10-E	Bio-TGAAAAATAAACTGTAAATCAAATTCCTC	gb|GQ396222.1|: bp72-44
	YR10-F	Bio-TGAAAAATAAACTGTAAATCATACTCCTC	gb|EU056622.1|: bp48-20
	YR10-G	Bio-TGAAAAATAAATTGTAAATCATACTCTTC	emb|HE805662.1|: bp124-96
	YR10-H	Bio-TGAAAAATAAATTGCAAATCATATTCTTC	gb|KC792556.1|: bp1133-1105
	YR10-I	Bio- TGAAAAATAAACTGTATGTCATATTCTTC	gb|JQ041819.1|: bp158-130
Probes for HPV16	Probe-16F	amine - TTTTTTTTTTTTTTTTAAGGAGTACCTACGACATGG	dbj|AB889494.1|: bp6718-6737
	Probe-16R	amine - TTTTTTTTTTTTTTTGATATGGCAGCACATAATGAC	dbj|AB889494.1|: bp6683-6663
Probes for HPV18	Probe-18F	amine - TTTTTTTTTTTTTTTTGCTTCTACACAGTCTCCTGTA	gb|KF225496.1|: bp239-259
	Probe-18R	amine - TTTTTTTTTTTTTTTCTTAAATTTGGTAGC ATCATATTG	gb|KF225496.1|: bp289-266
Probes for HPV31	Probe-31F	amine - TTTTTTTTTTTTTTTCCA CTCCATTTA AACCATCTG	gb|KC991270.1|:bp36-54
	Probe-31R	amine - TTTTTTTTTTTTTTTCGCCATGTCTTATAAATTGTT	gb|KC991270.1|: bp89-70
Probes for HPV33	Probe-33F	amine - TTTTTTTTTTTTTTTAT ATATAAGACATGTTGAAGAA	gb|KC706450.1|: bp265-244
	Probe-33R	amine - TTTTTTTTTTTTTTTCTGTCACTAGTTACTTGTGTGCAT	gb|KC706450.1|: bp293-361
Probes for HPV35	Probe-35F	amine - TTTTTTTTTTTTTTTCTGCTGTGTCTTCTAGTGACAG	gb|KC991278.1|: bp53-74
	Probe-35R	amine - TTTTTTTTTTTTTTTCACAGACATATTTGTACTACGGG	gb|KC991278.1|: bp48-26
Probes for HPV39	Probe-39F	amine - TTTTTTTTTTTTTTTTAGAGTCTTCCATACCTTCTAC	gb|KC470249.1|:bp6683-6703
	Probe-39R	amine - TTTTTTTTTTTTTTTGCCTGGTATATTCCTTAAACTTA	gb|KC470249.1|:bp6738-6716
Probes for HPV45	Probe 45F	amine - TTTTTTTTTTTTTTTTGACCCTACTAAGTTTAAGCAG	gb|KC470256.1|: bp6676-6697
	Probe-45R	amine - TTTTTTTTTTTTTTTGCACAGGATTTTGTGTAGAG	gb|KC470256.1|: bp6665-6646
Probes for HPV51	Probe-51F	amine - TTTTTTTTTTTTTTTTATTAGCACTGCCACTGCTG	gb|S40272.1|: bp16-36
	Probe-51R	amine - TTTTTTTTTTTTTTTCTTGGAGTAAATGTTGGGG	gb|S40272.1|: bp77-54
Probes for HPV52	Probe-52F	amine - TTTTTTTTTTTTTTTGGAATACCTTCGTCATGG	gb|KF225497.1|: bp987-1009
	Probe-52R	amine - TTTTTTTTTTTTTTTCCTTTTTAACCTCAGCAC	gb|KF225497.1|: bp1040-1018
Probes for HPV56	Probe-56F	amine - TTTTTTTTTTTTTTTTGACTATTAGTACTGCTACAGAA	gb|KC815983.1|: bp75-96
	Probe-56R	amine - TTTTTTTTTTTTTTTCG TGCATCATATTTACTTAACTG	gb|KC815983.1|: bp119-97
Probes for HPV58	Probe-58F	amine - TTTTTTTTTTTTTTGACATTATGCACTGAAGTAACTAAG	dbj|AB819279.1|: bp6668-6692
	Probe-58R	amine - TTTTTTTTTTTTTTTCATATTCCTTAAAATTATCATT	dbj|AB819279.1|: bp6729-6708
Probes for HPV59	Probe-59F	amine - TTTTTTTTTTTTTTTCCTAATGTATACACACCTACCAG	gb|KC470266.1|: bp6662-6684
	Probe-59R	amine - TTTTTTTTTTTTTTTAGAAGAAGTAGTAGAAGCACA	gb|KC470266.1|: bp6658-6638
Probes for HPV68	Probe-68F	amine - TTTTTTTTTTTTTTTCTACTACTGAATCAGCTGTACC	gb|KC470283.1|: bp6544-6565
	Probe-68R	amine - TTTTTTTTTTTTTTTCCTTAAATTTATTAGGATCATA	gb|KC470283.1|:bp6594-6573

For the establishment and validation of the MPHC assay, the HPV DNA was amplified with the YR8/YR10 primer set and the biotin-conjugated products were added to the probe-coated microwells. After alkaline denaturation, neutralization, hybridization and washing, horseradish-peroxidase-conjugated streptavidin (Dako) was used to bind target PCR products, and 3′,3′,5,5′-tetramethylbenzidine (Sigma-Aldrich) was then used to develop the color reaction. The optical density at 450 nm (OD_450_) of each well was measured using an enzyme-linked immunosorbent assay plate reader (SpectraMax M5).

The negative and positive cutoff values for the MPHC method were first estimated from 400 samples that were identified as negative by calculating the mean negative OD_450_ values plus two or three standard deviations to be 0.56 and 0.70, respectively (Fig. 1A). The cutoff values were then validated with another 100 specimens identified with the HCII assay (50 % negative and 50 % positive). It was found that the OD_450_ values of more than 95 % of the negative samples were lower than 0.56, and more than 95 % of the positive samples had values higher than 0.70 (Fig. 1B). Therefore, the equivocal zone was between 0.56 and 0.70. Ambiguous specimens were tested again in duplicate and were defined as positive when one result was positive or two results were equivocal in these duplicate reactions. Otherwise, when the value of a specimen that is suspected to be infectious is in the range of 0.70–1.0, we suggest that a second specimen be analyzed and/or an alternative testing method be used, especially for liquid-preserved cytology specimens.

**Fig. 1:**
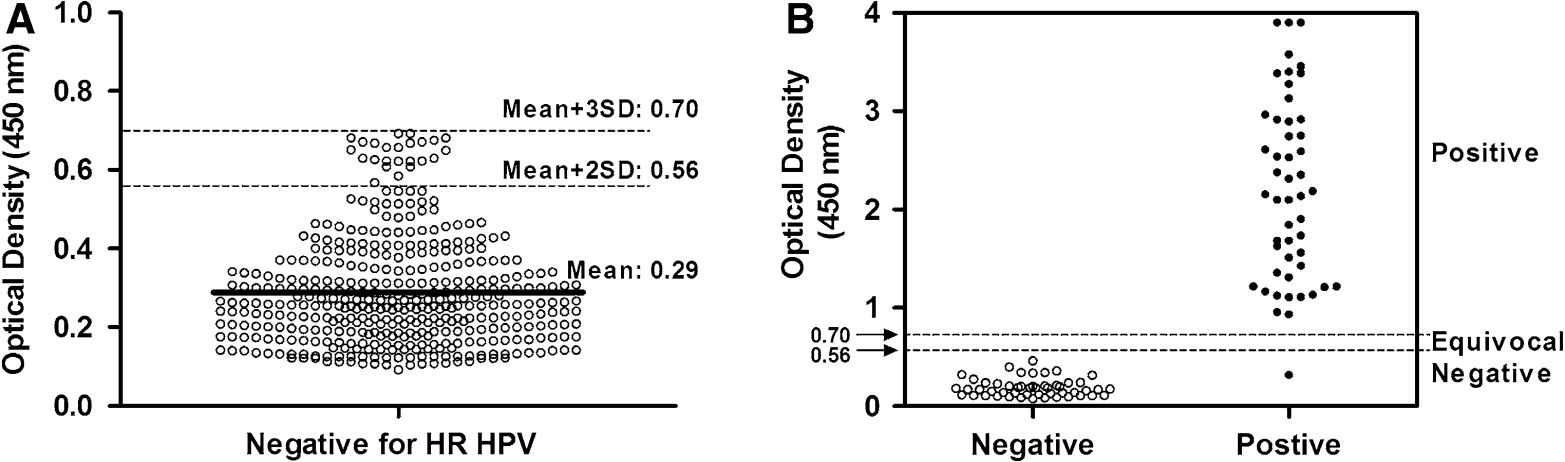
Determining the negative and positive cutoff values. **A**) Estimation of the cutoff value with 400 samples negative for HRHPV. The negative and positive cutoff values were estimated by calculating the mean negative OD_450_ values plus two or three standard deviations to be 0.56 and 0.70, respectively. **B**) Validation of the cutoff levels by MPHC assays performed on samples classified as negative or positive by the HCII assay. Using the cutoff value derived from the analysis described above as the criterion for positive specimens, more than 95 % of samples were identified as positive. The positive samples in the negative region were verified by DNA sequencing analysis and resolved as positive. This scatter plot was constructed with GraphPad Prism 5.0 (GraphPad Software, San Diego, CA, USA)

In order to determine the sensitivity and specificity of MPHC, 43 types of HPV (types 16, 18, 31, 33, 35, 39, 45, 51, 52, 56, 58, 59, 68, 6, 11, 26, 34, 40, 42, 43, 44, 53, 54, 55, 57, 62, 64, 66, 67, 69, 70, 71, 72, 73, 79, 80, 81, 82, 83, 84, 87, 90, and 91) plasmids were constructed. The sequence information for the HPVs was obtained from the GenBank database. To determine the detection limits of the MPHC for HPV DNA, a serial 10-fold dilution of each HRHPV plasmid and human genomic DNA from HPV18-transformed cells (PZ-HPV-7, ATCC CRL-2221D) was tested and analyzed using the MPHC assay to determine the positive cutoff concentration. To further determine the specificity of the assay, the assay was tested against DNA from viral strains, bacterial cultures, and clinical samples, including cytomegalovirus, herpes simplex virus 2, *Chlamydia trachomatis*, *Ureaplasma urealyticum*, *Neisseria gonorrhoeae*, *Mycoplasma humenis*, *M. genitalium*, *E. coli*, *Bacillus pyocyaneus*, *Staphylococcus aureus*, *Candida albicans*, and *Treponema pallidum*. The results showed that all 13 HRHPV plasmids were detected as positive at concentrations of 10^3^ copies/μL to 10^5^ copies/μL. At a concentration of 100 copies/μL, the OD values were close to the cutoff value for type 31 and type 52 and were 0.5–1.0 for type 56, but at a concentration of 500 copies/μL, they showed positive results. Only six types of plasmid (types 16, 18, 35, 45, 58, and 59) were positive at a concentration of 10 copies/μL (Fig. 2). At the concentration of 10^4^–10^5^ copies/μL, no positive results were obtained by the MPHC method for any of the non-HRHPV or other common genital-tract pathogens, indicating a lack of cross-reactivity between the high-risk and low-risk or genital-tract pathogens. It is believed that the specificity of the screen is determined only by the specificity of the probes. HPV18-transformed cells were used to compare the differences in the MPHC assay results when cells expressing virus or plasmids were analyzed. Although the sensitivity in detecting type 18 HRHPV was the same for the two samples, the HPV18-transformed cell based method was not suitable for evaluation of the differences in the primer and probe sequences between different HPV types.

**Fig. 2:**
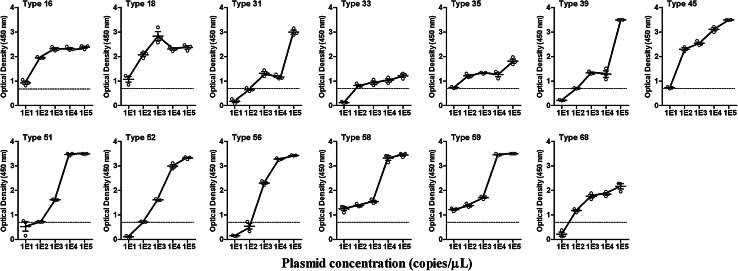
Detection limit of the MPHC assay for each of the 13 HRHPV plasmids at concentrations ranging from 10 copies/μL to 10^5^ copies/μL. The symbol (Ο) shows the OD_450_ value. The dotted line represents the positive cutoff value, estimated to be 0.7. Each data point represents the average of three duplicated experiments

To determine the epidemic levels of the 13 HRHPV types in high-risk populations in populous cities of China, 1238 cervical specimens were collected as described by the manufacturer of the HCII High-Risk HPV DNA test kit (Digene) [19] and detected by the MPHC and HCII method, respectively. The HCII test was performed with the automated HCII system as described previously [19]. Of the 1238 samples tested, 306 (25 %) were HRHPV positive and 862 (70 %) were HRHPV negative when measured by both methods. The two methods had a high concordance rate of 94.3 %. A kappa analysis showed a score of 0.859 (SPSS, ver. 18.0, IBM), indicating an “almost perfect” match between these two assays. However, the results for 70 (6 %) samples were discordant between the two assays. To confirm the type of virus present in the specimens with discordant results in HCII and MPHC, the samples were subjected to DNA sequencing, which showed that MPHC was correct for 33 samples, and HCII was correct for 6 samples. Twenty-seven specimens produced no HPV sequence results, and these were assumed to be HPV negative.

Based on these results, the sensitivity and specificity of the MPHC assay were 98.5 % (339/344) and 97.3 % (870/894), respectively. The HRHPV prevalence rate, when measured by MPHC, was 29.3 % (363/1238) in cervical specimens sampled from hospitals in three different regions of China. Although DNA sequencing is regarded as the gold-standard technique, there were still false negatives for the 24 samples diagnosed as positive by MPHC but negative by HCII assay (HCII–MPHC+), which were deemed to be negative based on sequencing failure, even if their MPHC detection values were more than 1.5. Because most sequencing failures are caused by the condition of the sample, including too little DNA, the 29.3 % positive detection rate with the MPHC method, which was higher than that with the HCII method, may be closer to the true positive rate. Therefore, the prevalence of HRHPV infection in female outpatients was between 27.8 % and 29.3 % in the regions of China examined, which was higher than those in the report by Li et al. [7].

The most prevalent HPV screening method is the HCII assay, which has been approved by the U.S. FDA. This method is validated by the strong agreement between its results and those of cytological or histological assays [20, 21]. However, the high cost of the assay makes it unfeasible for the routine mass screening of cervical infections in resource-poor areas of developing countries [22]. The present study suggested that the MPHC method is similar to HCII in terms of its specificity and sensitivity. In this study, using the same collection method, we eliminated the effects of the sample collection method. Therefore, the comparative results reflect the true efficiency of the PCR amplification, hybridization, and signal development achieved with the two methods.

A previous study revealed false positive results with the HCII method in detecting these 13 HRHPV types when 1 RLU/CO (relative light unit) was used as the cutoff value [23, 24]. The most frequently detected false positives occurred with HRHPV types 53, 66, 67, and 73 [25]. In this study, three of the five HCII-positive but MPHC-negative (HCII^+^MPHC^−^) samples were confirmed by sequencing to be true negatives, and they belonged to type 66, whereas the other two samples belonged to type 73. In the same analysis, three of the five HCII^+^MPHC^−^ samples were confirmed by sequencing to be true positives and belonging to type 52, whereas the other two samples were types 33 and 35. The sequencing results showed that the HPV types of the HCII^–^MPHC^+^ samples were limited to HPV types 16 (12/33), 58 (7/33), 59 (7/33), 35 (3/33), 45 (3/33) and 18 (1/33). These results suggested that the MPHC method was more sensitive than HCII in detecting HPV types 16, 35, 45, 58, and 59, and that the detection efficiency for type 52 requires further improvement. The MPHC assay for plasmids was slightly less sensitive in detecting three HPV types (types 31, 52, and 56) than in detecting the other types tested. The OD values at plasmid concentrations of 100 copies/μL were close to the cutoff value for type 31 and type 52 and were in the range of 0.5–1.0 for type 56. Therefore, their unstable concentration was 100 copies/μL. Despite finding no false-negative type 31 or type 56 HPV in the clinical samples (although we did find false-negative type 52), we must increase the sensitivity of their detection in this assay.

In summary, a sensitive, specific, low-cost, high-throughput PCR-based method was developed to detect 13 types of HRHPV in patient specimens. Although this hybridization process takes more time than the real-time PCR assay, it is a sensitive and specific assay that should enhance the study of HRHPV infections and the prevention of cervical cancer among a larger population. However, much effort should be made to standardize the established MPHC before it is widely applied clinically.

## Electronic supplementary material

Below is the link to the electronic supplementary material.

Supplementary material 1 (PDF 120 kb)

Supplementary material 2 (PDF 97 kb)
