# Supplementary material for: A PCR-based microwell-plate hybrid capture assay for high-risk human papillomavirus
Source: Arch Virol. 2014 Aug 5;159(12):3365–70. doi: 10.1007/s00705-014-2186-0 (PMC4221605; doi:10.1007/s00705-014-2186-0)
Supplement: Supplementary file 1 — Supplementary material 1 (PDF 120 kb) [file 705_2014_2186_MOESM1_ESM.pdf]

Professional English Writing and Editing

This certifies that the manuscript (with the title as “Development and Performance of a PCR-based Microwell-plate Hybrid Capture method for High-risk Human Papillomavirus”, Enuo case #: E20140119LQ) has been improved in grammar, punctuation, spelling, word usage, sentence structure, conciseness, general readability and writing style at Enuo Technology. Dr. Marc Chatenay-Lapointe, an associate editor at Enuo Technology, was in charge of this editing. Dr. Marc Chatenay-Lapointe who is a native English speaker got his Ph.D. degree from School of Medicine at Yale University. He has several scientific paper published on *Cell Metabolism* which has an impact factor of 17.7 in 2011.

Jian Liu, Ph.D.

Date: Feb 2, 2014

Managing Editor

Enuo Technology Inc.

Phone No.: +001-617-615-6758

Website: http://www.enuo1000.com

Email: service@enuo1000.com

Address: 59 Old Billerica Road

Bedford, MA, 01730, United States
