# Supplementary material for: A PCR-based microwell-plate hybrid capture assay for high-risk human papillomavirus
Source: Arch Virol. 2014 Aug 5;159(12):3365–70. doi: 10.1007/s00705-014-2186-0 (PMC4221605; doi:10.1007/s00705-014-2186-0)
Supplement: Supplementary file 2 — Supplementary material 2 (PDF 97 kb) [file 705_2014_2186_MOESM2_ESM.pdf]

Certificate of English Editing

**Date of Issue**	13 May 2014

About the manuscript:

**Title**	A PCR-based microwell-plate hybrid capture assay for high-risk human papillomavirus
**First Author**	Yumei Wang
**Affiliation**	Department of In Vitro Diagnostic Reagents, Key Laboratory of the Ministry of Health for Research on Quality and Standardization of Biotech Products, National Institutes for Food and Drug Control, Beijing 100050, China. No. 2 Tiantanxili, Beijing 100050, China.
**Date of editing**	13 May 2014

About the editor:

Editor

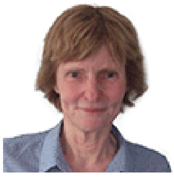

Janine Miller

1999 - PhD Molecular Biology - University of Tasmania

An experienced molecular biologist with more than 25 years of experience in research, editing, and teaching science

Full profile

**Certificate issued by**	Benjamin Shaw<br>Director<br><br>Liwen Bianji (Edanz Group China)

Liwen Bianji (Edanz Group China)
Interchina Commercial Building, 1112A
No 33. Dengshikou Street, Dongcheng District, Beijing, P.C. 100006, China
Phone +86-10-6528-0877 Fax +86-10-6528-0834 Email editing@liwenbianji.cn
